# Gender-Related Difference in Skin Oxygenation in Young Patients with Uncomplicated Type 1 Diabetes

**DOI:** 10.3390/biomedicines12071413

**Published:** 2024-06-25

**Authors:** Jolanta Neubauer-Geryk, Małgorzata Myśliwiec, Leszek Bieniaszewski

**Affiliations:** 1 Clinical Physiology Unit, Medical Simulation Centre, Medical University of Gdańsk, 80-210 Gdańsk, Poland; lbien@gumed.edu.pl; 2 Department of Pediatrics, Diabetology and Endocrinology, Medical University of Gdańsk, 80-211 Gdańsk, Poland; malgorzata.mysliwiec@gumed.edu.pl

**Keywords:** gender, type 1 diabetes mellitus, skin microcirculation, capillaroscopy, transcutaneous oxygen pressure, children and adolescents

## Abstract

Gender, through genetic, epigenetic and hormonal regulation, is an important modifier of the physiological mechanisms and clinical course of diseases. In diabetes mellitus, there are gender differences in incidence, prevalence, morbidity, and mortality. This disease also has an impact on the microvascular function. Therefore, this cross-sectional study was designed to investigate how gender affects the cutaneous microcirculation. We hypothesized that gender should be an important factor in the interpretation of capillaroscopy and transcutaneous oxygen saturation results. The study group consisted of 42 boys and 55 girls, uncomplicated diabetic pediatric patients. Females (F) and males (M) did not differ in terms of age, age at onset of diabetes, or diabetes duration. Furthermore, they did not differ in metabolic parameters. The comparison showed that group F had lower BP, higher pulse, and higher HR than group M. Group F had significantly lower creatinine and hemoglobin levels than group M. In children and adolescents with type 1 diabetes without complications, there was a gender difference in microcirculatory parameters. The resting transcutaneous partial pressure of oxygen was significantly higher in females than in males. However, there were no gender-related differences in basal capillaroscopic parameters or vascular reactivity during the PORH test. Our results indicate that studies investigating the structure and function of the microcirculation should consider the role of gender in addition to known cofactors such as puberty, body mass index, physical activity, and cigarette smoking.

## 1. Introduction

The pressure-dependent myogenic constriction of arterioles [1] is considered a crucial mechanism for homeostasis. In animal studies, it was shown that the myogenic constriction of arterioles in female rats was less pronounced than in male rats due to the increased release and activity of NO (nitric oxide) associated with the presence of estrogen [2].

In the general population, estrogens appear to be protective against CVD [3,4] before menopause. Estrogens bind to estrogen receptors alpha and beta and to G protein-coupled receptors, exert both genomic and extragenomic effects, and their expression in arteries is higher in women than in men, but decreases with menopause [5].

The result of nongenomic signals is the rapid activation of endothelial nitric oxide synthase and the production of nitric oxide, which promotes vasodilation [6]. Furthermore, estrogen has anti-proliferative effects on vascular smooth muscle cells, promotes re-endothelialization, reduces the pro-inflammatory activation of vascular endothelial cells, and modulates the production of reactive oxygen species [7]. Estrogens improve the lipid profile [8] and also increase the visceral fat, causing visceral obesity followed by metabolic syndrome [9]. In addition, traditional risk factors such as elevated blood pressure, smoking, overweight and obesity, diabetes, and elevated cholesterol are significant for both genders, but there are differences in their magnitude [10]. The worse prognosis in women may be due to disorders of the hypothalamic–pituitary–ovarian axis [11]. Compared to women without diabetes, adolescent girls with T1D have been shown to have lower estradiol levels [12,13]. This may contribute to their adverse metabolic profile, a more atherogenic lipid profile, insulin resistance, greater inflammation, and the loss of vasoprotective effects. During adolescence, male children develop more lean body mass. In contrast, female children gain more fat mass, a physiological change that may be exacerbated in type 1 diabetes by the amount of insulin taken [14].

Studies in humans have shown that the sensitivity and/or density of peripheral vascular adrenergic receptors is lower in females than in males, so that alpha-adrenergic vasoconstriction is lower in young females than in males [15,16]. There are differences in beta-adrenergic receptors. Beta-adrenergic receptors compensate for α-adrenergic vasoconstriction in young women but not in young men or postmenopausal women [17]. In the cerebrovascular system, gender differences have been described in vascular anatomy [18] and pharmacology [19].

Gender is an important modifier of physiological mechanisms and the clinical course of diseases through genetic, epigenetic, and hormonal regulation. Diabetes, hypertension, and heart failure show gender differences in incidence, prevalence, morbidity, and mortality. These diseases also affect the microvascular function [20,21,22,23]. It is well known that coronary heart disease (CHD) and their symptoms vary between men and women. In men, it occurs at a younger age. It is a macrovascular disease characterized by the presence of coronary obstruction and the deposition of atherosclerotic plaques. In contrast, CHD in women is a microvascular disease manifested by increased arteriolar narrowing and vasospasm [24]. A U.K. study published in late 2023 found significant gender differences in the coronary microvascular response to myocardial ischemia–reperfusion, which may explain why some studies have reported worse outcomes in women after myocardial infarction [25].

The cutaneous microcirculation may be studied with the use of a wide spectrum of measurement methods [26,27,28]. In clinical practice, capillaroscopy and transcutaneous oxygen pressure (TcPO_2_) are widely used. The first one gives insights into the structure and function of the microcirculation with the use of provocative tests. This technique is often used in studying the cutaneous microcirculation in a wide spectrum of diseases, e.g., peripheral vascular disease [29], type 1 diabetes [30,31,32,33,34,35,36,37], hypertension [38], type 2 diabetes [39,40], or obesity [41].

The TcPO_2_ method provides the most direct functional microcirculatory assessment by measuring tissue oxygenation, which can be affected by many factors such as blood flow [42], arterial oxygen pressure [43], rheological parameters [44,45], and arteriovenous fistulas [46]. In recent years, the practical value of tcPO_2_ in the screening of vascular disease has been well documented [47,48], as well as its use in the assessment of wound healing progress [49], the efficacy of revascularization procedures [50], the prediction of amputation rates [49], the durability of skin grafts performed [51], and the qualification for oxygen therapy in a hyperbaric chamber [51].

In previous reports, we presented the results of our studies on the structure and function of the cutaneous microcirculation with the use of capillaroscopy and transcutaneous oxygen pressure (TcPO_2_) [26,31,52,53]. In the study of microcirculation in diabetic patients without microangiopathy, it is important to be aware of possible physiological differences. The determination of the dependence of the capillaroscopy or transcutaneous measurement of oxygen partial pressure parameters on anthropometric, hemodynamic, or laboratory indicators will allow for approaching the establishment of reference values for these methods. Therefore, the aim of this study was to analyze the impact of gender on cutaneous microcirculation parameters. We hypothesized that gender should be considered as an important factor in the interpretation of capillaroscopy and transcutaneous oxygen saturation results.

## 2. Materials and Methods

### 2.1. The Study Group

The study group included 97 patients with type 1 diabetes (T1D), 42 boys and 55 girls, with a mean age of 15.4 (8.4–20.1) and 14.9 (11.5–19.1) years, respectively, who were treated at the Department of Pediatrics, Diabetology, and Endocrinology, University Clinical Center, in Gdańsk. The patients met the diagnostic criteria for type 1 diabetes according to the International Society of Child and Adolescent Diabetes [54]. The mean age at the onset of diabetes was 10.2 and 9.4 years, and the mean diabetes duration was 4.8 and 5.5 years, respectively, for males and females. The Tanner stage was used to assess puberty [55,56]. It identifies five specific stages of physical change during puberty, including developing genitals, breasts, and pubic hair. Tanner stage 1 corresponds to the pre-pubertal stage, with a progression to Tanner stage 5, which is the final adult form.

The study exclusion criteria were micro- and macroangiopathic complications, acute complications of diabetes, abnormal TSH and free thyroxine levels, autoimmune thyroiditis, systemic diseases like rheumatoid arthritis and psoriasis, and statin use. Diabetic retinopathy was excluded based on an ophthalmoscopic evaluation of the fundus after pupil dilation by an ophthalmologist according to the American Diabetes Association criteria [57]. The diagnosis of diabetic neuropathy was based on subjective and objective neuropathy symptoms [58]. Diabetic nephropathy was diagnosed based on twice demonstrated albuminuria >30 mg/day in the past 6 months.

Severe hypoglycemia was classified as an episode of blood glucose <54 mg/dL requiring intervention by another person that occurred within one year before the survey but no more than one month before the survey. Mild hypoglycemia was defined as a non-interventional glucose episode <54 mg/dL in the month before the survey [57].

A physical investigation was performed by an experienced pediatrician. Informed consent was obtained from the subjects after a detailed explanation of the study’s purpose and conduct. The parents provided their consent to the study and were present during the study.

The applied research methodology was approved by the Independent Bioethics Committee for Scientific Research at the Medical University of Gdańsk (decisions NKBBN/277/2014 (Independent Bioethics Committee for Research) of 8 July 2014 and NKBBN/277-512/2016 of 5 December 2016).

Prior to the capillaroscopic examination, the resting systolic and diastolic blood pressures were measured five times using an OMRON HEM-907 automatic sphygmomanometer (OMRON Healthcare Europe B.V., Hoofddorp, The Netherlands) in a sitting position after resting for at least 10 min. Blood pressure and heart rate were measured according to the “Pediatric Primary Hypertension: An Underrecognized Condition: A Scientific Statement From the American Heart Association” [59]. Pulse pressure was calculated as the difference between systolic and diastolic pulse pressures.

### 2.2. Evaluation of Microcirculation

The patients were asked to refrain from undergoing any cosmetic procedures for at least 2 weeks prior to testing. Before the study, the patients were asked to avoid finger cosmetic procedures for 2 weeks. Acclimatization to the room temperature of 20 °C took place for 15 min prior to the start of the study, which allowed for an adequate thermal adaptation. Body temperature was monitored using a noncontact thermographic device (Novama model NT-19, AVITA Corporation, MDSS GmbH, Hannover, Germany) and was within the normal range in all subjects.

#### 2.2.1. Capillaroscopy Examination

Capillaroscopy was performed according to a procedure we described many times previously [26,31,34,37,53]. In brief, capillaroscopy was performed for the evaluation of the nail bed capillaries of fingers II–V of both upper limbs. Before capillaroscopy, the selected fingernail was cleaned and covered with immersion oil to optimize image quality. Capillaroscopy was performed in a seated position with the hands supported, so that the hands could rest freely under the capillaroscope. The procedure was performed with an OPTA-TECH two-point illumination capillaroscope with a digital 5-megapixel camera at 200× magnification. Imaging was recorded on a disc using the manufacturer’s standard software (Multiscan Base v18.03, OPTA-TECH, Warsaw, Poland).

The capillaroscopic images were processed according to a method developed in the Clinical Physiology laboratory. This method was published in Diabetes Care in 2013 [31]. Capillaroscopy was then repeated twice: after 20 min of rest in the sitting position and after 4 min of active occlusion induced by arm compression with a sphygmomanometer cuff at a pressure 50 mmHg higher than the patient’s systolic pressure. As parameters characterizing the skin microcirculation, the mean distance between successive capillaries (Distance), the ratio between the area covered by capillaries, and the total area of the analyzed capillary rows (Coverage) were determined. These indices were obtained under baseline conditions and after the PORH test (post-occlusive reactive hyperemia). Microcirculatory reactivity was assessed as capillary reactivity, understood as the quotient of the difference between post-PORH and pre-PORH coverage to baseline coverage, and as the coverage_ratio, calculated as the percentage change in coverage value in the PORH test.

#### 2.2.2. Transcutaneous Oxygen Pressure Measurement

Transcutaneous oxygen pressure was assessed on the patient’s forearm. The sensor was placed in the middle part of the forearm, cleaned and hairless. The transcutaneous oxygen test used the PeriFlux 5000 instrument (Perimed AB, Järfälla, Sweden). TcPO_2_ is based on the amount of oxygen that diffuses from the capillaries through the epidermis to the electrode and provides information about the body’s ability to deliver oxygen to the tissues [60,61]. The principle of measuring the transcutaneous partial pressure of oxygen was presented in detail previously [26,37,52].

TcPO_2_ was recorded after resting for at least 20 min, during occlusion, and after deflating the cuff. The curve analysis involved identifying specific points, i.e., T_base for the moment when occlusion began, T_zero for the moment when occlusion ended, and TTR (time to reach the baseline value after occlusion) for the moment when recovery was achieved.

### 2.3. Laboratory Analysis

Blood samples were taken between 7 and 9 am after an overnight fast. For all measurements, the same blood sample was used. HbA_1c_, with a normal range of 3.0 to 6.0%, was measured by an immunoturbidometric method using the Unimate 3 set. The total cholesterol, HDL (high-density lipoproteins), LDL (low-density lipoproteins), and triglyceride levels were measured using Cormay enzymatic kits. The CREA assay system was used to measure the serum creatinine levels. TSH (thyroid-stimulating hormone) and fT4 (free thyroxine) were measured using a heterogeneous immunochemiluminescence assay. All measurements were performed in the ISO-accredited laboratory of the University Clinical Center.

### 2.4. Statistics

The statistical analysis of the data obtained was performed using SAS^®^ OnDemand for Academics, SAS Institute Inc., SAS Campus Drive, Cary, NC 27513, U.S.A. The distribution of the variables was assessed using the Shapiro–Wilk test. The Mann–Whitney test was used to compare groups. The chi-square test was used to compare gender proportions, Tanner stages, and hypoglycemic episodes.

The Spearman correlation coefficients of the relationship between TcPO_2__base and the variables that differed between the two groups and between these variables were determined. 

Comparisons between groups were performed using the general linear model with Fisher’s post hoc test when needed. The influence of covariates was studied using the ANCOVA procedure. A significance level of *p* < 0.05 was considered statistically significant.

## 3. Results

The studied groups did not differ in anthropometric parameters, age, age of disease onset, or disease duration (Table 1). Girls (group F) and boys (group M) used similar insulin doses. There were no statistical differences in the duration of the insulin pump treatment or the number of all hypoglycemic events.

A comparison analysis showed that the F-group had lower systolic blood pressure (*p* = 0.01) and pulse pressure (*p* = 0.009) and higher heart rate (*p* = 0.001) than the M-group.

**Figure 1:**
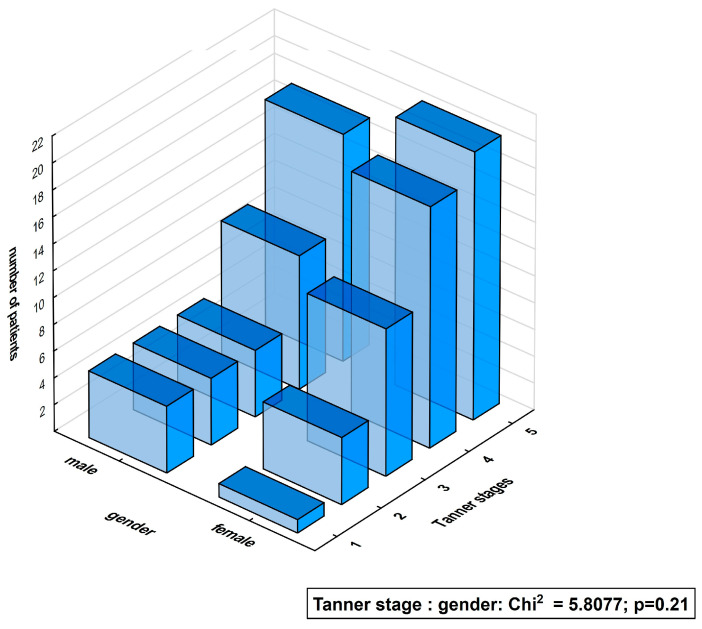
Distribution of Tanner sexual gender maturity stages (1–5) in type 1 diabetes males and females.

There was no difference between girls and boys in the percentage of subjects in subsequent Tanner sexual gender maturity stages (Figure 1).

Metabolic parameters such as lipidogram and current HbA_1c_ levels did not differ between the subgroups, nor did the thyroid hormone levels (Table 2). The F-group had significantly lower creatinine levels (*p* = 0.004) compared to the M-group. Analysis of erythrocyte parameters showed significantly higher hemoglobin (*p* < 0.001) levels in group M than in group F (Table 2).

**Figure 2:**
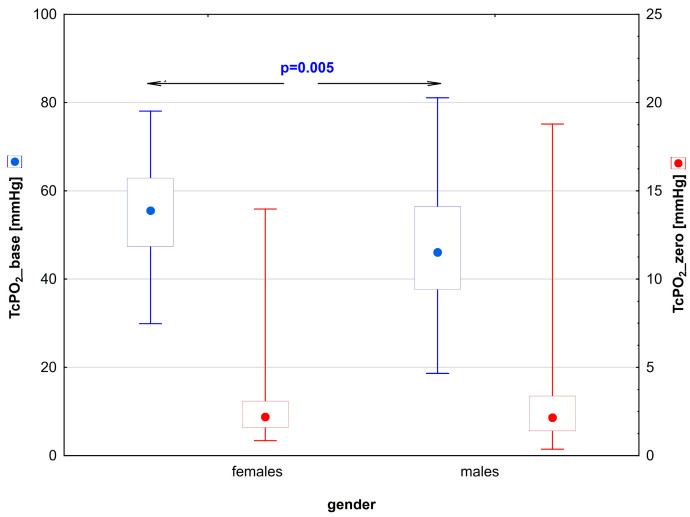
Comparison of the transcutaneous oxygen parameters TcPO_2__base and TcPO_2__zero in young patients with type 1 diabetes divided by gender. The red and blue dots indicate the median for the respective variable. The value of *p* < 0.05 was regarded as statistically significant.

It was shown that the capillaroscopic parameters did not differ between the sexes both at the beginning of the test and after the PORH test (Table 3). However, significant differences were found in the parameters that describe the transcutaneous pressure of oxygen. The baseline value of TcP0_2_ (TcPO_2__base) was significantly higher in women than in men. The biological zero value (TcPO_2__zero) was comparable in both genders (Figure 2, Table 3).

**Figure 3:**
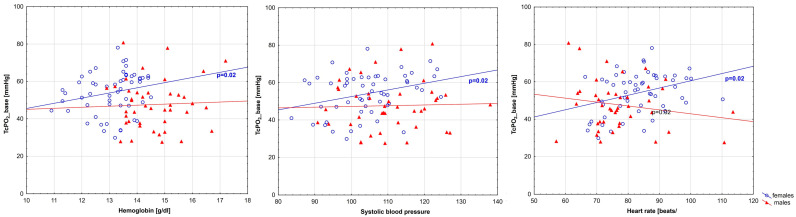
Scatterplots illustrating the relationship between TcPO_2__base (TcPO_2__base—mean value of TcPO_2_ within 60 s before PORH) and systolic blood pressure (left plot), hemoglobin level (middle plot), and heart rate (right plot) in females and males. The value of *p* < 0.05 was regarded as statistically significant. The red triangles and blue circles indicate the median for the respective variable.

The correlation analysis between TcPO_2_ and the parameters that differed between the subgroups of girls and boys showed a significant correlation with hemoglobin concentration, systolic blood pressure level, and heart rate, but only in the female group. In the male group, there was no correlation between TcPO_2_ and any of the parameters (Table 4, Figure 3).

Analyzing the relationships between systolic blood pressure, heart rate, pulse pressure, and hemoglobin showed (Table 5) that some sets of parameters could be introduced into the GLM analysis, as reported in Table 6.

The higher level of TcPO_2_ in the group of girls persisted even after adjustment for the variables that distinguished the subgroups (Table 6).

## 4. Discussion

Differences in parameters describing the microcirculation may result from innate physiological differences [62] between men and women. The present study of the cutaneous microcirculation employing capillaroscopy and the transcutaneous measurement of the partial pressure of oxygen and the PORH test showed that there was a gender-related difference in the microcirculatory parameters in children and adolescents with type 1 diabetes without complications. Females were found to have a significantly higher resting transcutaneous oxygen partial pressure compared to males. However, there were no gender differences in the capillaroscopic parameters under basal conditions or in the reactivity of the microcirculation in the PORH test.

Numerous studies have highlighted the influence of gender-related hormones on microcirculation reactivity, contributing valuable insights to our understanding of this issue [63,64,65,66]. Vasodilation is caused by estrogen through both a large increase in nitric oxide (NO) production and the induction of nitric oxide synthase (NOS) genes. Long-term estrogen therapy reverses the abnormal endothelial function associated with menopause [64]. In male rats with liver ischemia/reperfusion, some microvascular damage was reversed when the rats were treated with estrogen before ischemia [63]. On the other hand, the effect of testosterone is unclear, as this hormone may improve [65] or worsen [66] endothelial dysfunction. In addition, androgens may play an important role in the gender differences in the regulation of blood pressure. Research findings from ambulatory blood pressure monitoring in children suggest that the blood pressure levels tend to increase with age in both boys and girls. However, after the onset of puberty, the systolic blood pressure was higher in boys than in girls [66,67]. In our study, we found that females had lower systolic blood pressure and a higher heart rate. However, these physiological differences did not seem to influence the gender disparity in basal transcutaneous oxygen pressure.

Women may experience regular blood loss, leading to significantly lower hemoglobin levels compared to men [68]. However, it is important to note that despite these lower hemoglobin levels, our results showed that females had significantly higher basal oxygen concentrations. There are some well-described physiological adaptations to anemia in women, including a rightward shift in the hemoglobin–oxygen dissociation curve to reduce the affinity for oxygen secondary to higher levels of 2,3-diphosphoglycerate.

In addition to its vasodilatory effect on the smooth muscle cells and endothelial cells of the vasculature leading to lower blood pressure [69], estrogen also affects heat loss [70]. The microcirculation in the skin is directly related to the temperature of the skin. In our study, the skin temperature was controlled by a thermal electrode and kept at 43 °C.

The results of published reports on gender impact on microcirculation studied by capillaroscopy are difficult to compare with the results obtained in our study because of differences in the studied subpopulations of subjects and in the techniques used. In the group of young people with T1D that we studied, we found no differences in parameters such as coverage and distance both in basal condition and after PORH. The available studies were conducted on a general healthy population and focused on nail capillary structure [71,72]. They showed that male participants had lower subpapillary venous plexus visibility scores, but other capillary parameters did not differ between the sexes. Similarly, no gender differences in capillaroscopic parameters were found in a study by Bogusz-Górna et al. in a group of children and adolescents with type 1 diabetes [73]. It should be noted that in these studies, a qualitative approach in analyzing the capillaroscopy results was used, while we had the advantage of performing a quantitative description of the capillaries. Also in another study of healthy volunteers applying confocal laser scanning microscopy, no gender-related differences were identified regarding such capillary parameters as area, perimeter, circularity, and maximum diameter [74].

An important feature of the microcirculation is its reactivity to ischemia. In our study, we found that there was no difference between the sexes regarding the parameters derived from capillaroscopy (coverage_ratio) as well as based on the measurement of transcutaneous oxygen pressure (TTR) during PORH.

Similar results were obtained in the study of Heimhalt-El Hamriti et al. for groups of healthy children and age-matched diabetic patients, with a duration of 5.0 ± 3.97 years. LDF (Laser Doppler Fluxometry) and stimuli other than PORH were used [75]. In this report, researchers found that none of the flow parameters were associated with gender. In addition, in both patients and healthy subjects, the stage of sexual maturation did not influence the response to the applied stimuli.

A gender-related difference in microvascular reactivity was found by Stupin et al. [76]. They found that healthy young women had higher skin microvascular reactivity than men in the LDF and PORH tests. Women also had lower systolic blood pressure and heart rate. However, women in this study had lower body mass index and waist-to-hip ratio and higher body fat mass than men [76].

Many researchers have used tcPO_2_ to evaluate the microvascular function [77,78,79,80]. In the present study, we reported and analyzed the confounders for the gender-related difference in tissue oxygen concentration as measured by tcPO_2_ in standardized resting conditions. We found that females had significantly higher tcPO_2_ levels than males, despite other factors that could potentially interact with tcPO_2_. Orenstein et al. study [77] in healthy volunteers showed that women had a significantly higher tcPO_2_ than men. In an interesting work by Dooley et al., 72 healthy men and women breathed air and 10% O_2_ serially. The researchers found that tcPO_2_ measured in various areas of the body was higher in females than in males [78]. Rodrigues et al. [79] in their comparison of tcPO_2_ between female and male groups found no gender-related differences in tcPO_2_. In a study by Dowd et al. of a group of 205 volunteers, the authors did not find a significant correlation between gender and the magnitude of the transcutaneous partial pressure of oxygen [80].

Cigarette smoking is known to induce microvascular dysfunction in the skin, and the severity of this impairment is known to be independently related to the duration and intensity of exposure to cigarette smoke. Therefore, smoking patients were excluded from the study [81]. A limitation of our work may be the lack of assessing the mass composition, especially muscle mass, which does not allow us to compare our results with other works. In addition, we based the degree of sexual maturity only on the Tanner scale and we did not examine the hormonal levels of estrogen and testosterone. The absence of microangiopathic complications in children and adolescents with diabetes in this study is both a limitation and a strength. We also had no opportunity to study patients with longer diabetes duration. Although our patients did not present signs of infection, it would have been worthwhile to evaluate parameters of inflammation that may have an impact on microcirculatory parameters.

Repeated exercise has strong and independent beneficial effects on the cardiovascular system [82]; moreover, physical exertion alters the cutaneous blood flow. Regularly trained individuals have higher core temperature and cutaneous blood flow at all levels of exercise [82]. Furthermore, repeated physical activity increased the responsiveness of the skin microcirculation to several vasodilator stimuli [26,28,83]. In our group, we did not collect any information on the type of physical activity and the intensity of physical activity performed by the patients.

## 5. Conclusions

We observed a significant effect of gender on transcutaneous oxygen concentration in young patients with type 1 diabetes. The obtained result suggests the importance of considering gender when analyzing microcirculatory studies.

## Figures and Tables

**Table 1:** Characteristics of all diabetic patients enrolled in the study divided into females and males. Data are presented as median (range) or mean ± SD.

Characteristics	Diabetic Patients’ Subgroups According to Gender		
	Females (F), *n*= 55	*p* for between-Group Comparison	Males (M), *n* = 42
Age [years]	14.9 (11.5–19.1) 15.2 ± 2.1	0.83	15.4 (8.4–20.1) 15.2 ± 2.6
Onset of diabetes [age]	9.4 (2.1–14.9) 9.3 ± 3.4	0.92	10.2 (1.2–15.0) 9.2 ± 4
T1D duration [years]	5.5 (0.6–13.1) 5.9 ± 3.4	0.96	4.8 (0.3–14.5) 6.1 ± 4
Body mass [kg]	56.8 (37.8–95.7)	0.13	62.2 (28.8–100)
BMI [kg/m^2^]	20.5 (15.7–29.7)	0.29	20.3 (14.5–28.3)
Insulin dose [units/24 h]	45 (10.3–75)	0.52	45 (1.5–100)
Time of pump treatment as ratio to T1D duration [%]	50 (0–100)	0.85	36 (0–100)
HbA_1c_ [%]	7.8 (5.3–13.6)	0.93	7.6 (5.5–11.3)
Episodes of mild hypoglycemia [N/last month]	6 (0–20)	0.92	5.5 (0–30)
Episodes of severe hypoglycemia [N/last year]	0 (0–2)	0.57	0 (0–3)
Systolic blood pressure [mmHg]	104 (84–125)	0.01	109 (91–138)
Diastolic blood pressure [mmHg]	60 (49–76)	0.65	61 (49–72)
Heart rate [beats/min.]	83 (66–110)	0.001	76 (57–114)
Pulse pressure [mmHg]	44 (27–60)	0.009	49.3 (33.2–69.6)

Abbreviations: HbA_1c_—glycated hemoglobin; BMI—body mass index; T1D—diabetes mellitus. The value of *p* < 0.05 was regarded as statistically significant.

**Table 2:** Comparison of laboratory results in diabetic patients divided into the subgroups of females and males (F, M). Data are presented as median (range).

Characteristics	Diabetic Patients’ Subgroups According to Gender		
	Females (F), *n* = 55	*p* for between-Group Comparison	Males (M), *n* = 42
Total cholesterol [mg/dL]	179 (114–288)	0.35	173 (119–246)
Cholesterol LDL [mg/dL]	102 (57–188)	0.66	100 (61–164)
Cholesterol HDL [mg/dL]	57 (33–120)	0.22	54 (35–90)
Triglycerides [mg/dL]	69 (34–294)	0.76	75 (38–154)
TSH [mIU/L]	1.6 (0.6–4.2)	0.18	1.9 (0.6–5.1)
fT4 [pmol/L]	12.4 (9.3–14.5)	0.28	12.8 (9.0–18.1)
Serum creatinine [mg/dL]	0.7 (0.5–0.9)	0.002	0.8 (0.5–1.2)
Hemoglobin [g/dL]	13.4 (10.9–14.5)	<0.001	14.5 (12.9–17.2)

Abbreviations: TSH—Thyroid-stimulating hormone; fT4—free thyroxine; LDL—low-density lipoproteins; HDL—high-density lipoproteins. The value of *p* < 0.05 was regarded as statistically significant.

**Table 3:** Comparison of microcirculation parameters in diabetic patients divided into the subgroups of females and males (F, M). Data are presented as median (range).

Characteristics	Diabetic Patients’ Subgroups According to Gender		
	Females (F), *n* = 55	*p* for between-Group Comparison	Males (M), *n* = 42
Capillaroscopy			
Coverage_BASE_ [%]	17.4 (12.7–23.6)	0.50	17.4 (11.5–24.8)
Coverage_PORH_ [%]	16.2 (10.4–24.3)	0.45	15.9 (9.8–24.4)
∆Coverage_PB_ [%]	−0.7 (–8–4.9)	1	−1.0 (–5.8–3.3)
Capillary reactivity	−4.8 (–41–31)	0.99	−6.0 (–35.1–19.5)
Coverage_ratio [%]	95 (59–131)	0.99	94 (65–119)
Distance_BASE_ [µm]	224.1 (165.8–307.3)	0.71	225.5 (179.4–377.7)
Distance_PORH_ [µm]	225.5 (166.3–334.2)	0.48	234.8 (180.5–345.1)
∆Distance_PB_ [µm]	6.91 (−63.3–105.8)	0.97	10.4 (−70.4–90.5)
Transcutaneous oxygen pressure			
TcPO_2__base [mmHg]	56 (29.9–78.1)	0.005	46.5 (27.6–80.8)
TcPO_2__zero [mmHg]	2.4 (0.9–13.7)	0.81	2.8 (0.7–18.8)
TTR [s]	85 (28–240)	0.85	80.5 (32–240)

Abbreviations: PORH—post-reactive hyperemia; Coverage_BASE_—ratio of the capillary area to the total area of the determined rows in baseline condition; Coverage_PORH_—ratio of the capillary area to the total area of the determined rows after PORH; ∆Coverage_PB_ = Coverage_PORH_–Coverage_BASE_; Capillary reactivity = ∆Coverage_PB_/Coverage_BASE_; Coverage_ratio—percentage change in the coverage value in the PORH test; Distance_BASE_—mean distance between successive capillaries in baseline condition; Distance_PORH_—average distance between successive capillaries after PORH; ∆Distance_PB_ = Distance_PORH_–Distance_BASE_; TcPO_2__base—mean value of TcPO_2_ within 60 s before T_base; TcPO_2__zero–mean value of TcPO_2_ within 60 s before T_zero; TTR—time to reach the baseline value after occlusion. The value of *p* < 0.05 was regarded as statistically significant.

**Table 4:** Spearman’s correlation coefficients between TcPO_2__base and parameters differing between males and females.

Parameter	Females		Males	
	r	*p*	r	*p*
Hemoglobin	0.30	0.02	−0.01	0.95
Serum creatinine	−0.20	0.14	−0.02	0.89
Systolic blood pressure	0.30	0.02	−0.00	1.00
Heart rate	0.32	0.02	−0.11	0.48
Pulse pressure	0.21	0.12	−0.04	0.79

The value of *p* < 0.05 was regarded as statistically significant.

**Table 5:** Spearman’s correlation coefficients between parameters differing between the groups of males and females and correlating with TcPO_2__base.

Parameter	Females						Males					
	Systolic Blood Pressure		Heart Rate		Pulse Pressure		Systolic Blood Pressure		Heart Rate		Pulse Pressure	
	r	*p*	r	*p*	r	*p*	r	*p*	r	*p*	r	*p*
Hemoglobin	0.07	0.59	0.26	0.06	−0.20	0.15	0.37	0.02	−0.30	0.06	0.25	0.1
Systolic blood pressure			0.41	0.002	0.77	<0.001			−0.20	0.21	0.84	<0.001
Heart rate					−0.01	0.96					−0.44	0.004

The value of *p* < 0.05 was regarded as statistically significant.

**Table 6:** Characteristics of TcPO_2__base in the studied groups. Values are presented as median and range.

Characteristics		Diabetic Patients’ Subgroups According to Gender		*p* for between-Group Comparison
		Females, *n* = 55	Males, *n* = 42	
TcPO_2__base [mmHg]		56 (29.9–78.1)	46.5 (27.6–80.8)	0.017
After adjustment for hemoglobin				0.01
	Systolic blood pressure			0.003
	Heart rate			0.02
	Hemoglobin and systolic blood pressure			0.004
	Hemoglobin and heart rate			0.03

The value of *p* < 0.05 was regarded as statistically significant. Abbreviations: TcPO_2__base—mean value of TcPO_2_ within 60 s before PORH.

## Data Availability

The research data can be requested from the first author.

## References

[1] Levy B.I., Ambrosio G., Pries A.R., Struijker-Boudier H.A.J. (2001). Microcirculation in Hypertension: A New Target for Treatment?. Circulation.

[2] Huang A., Sun D., Koller A., Kaley G. (1997). Gender Difference in Myogenic Tone of Rat Arterioles Is Due to Estrogen-Induced, Enhanced Release of NO. Am. J. Physiol. Circ. Physiol..

[3] Walli-Attaei M., Joseph P., Rosengren A., Chow C.K., Rangarajan S., Lear S.A., AlHabib K.F., Davletov K., Dans A., Lanas F. (2020). Variations between Women and Men in Risk Factors, Treatments, Cardiovascular Disease Incidence, and Death in 27 High-Income, Middle-Income, and Low-Income Countries (PURE): A Prospective Cohort Study. Lancet.

[4] Maas A.H.E.M., Rosano G., Cifkova R., Chieffo A., van Dijken D., Hamoda H., Kunadian V., Laan E., Lambrinoudaki I., Maclaran K. (2021). Cardiovascular Health after Menopause Transition, Pregnancy Disorders, and Other Gynaecologic Conditions: A Consensus Document from European Cardiologists, Gynaecologists, and Endocrinologists. Eur. Heart J..

[5] Vitale C., Mendelsohn M.E., Rosano G.M.C. (2009). Gender Differences in the Cardiovascular Effect of Sex Hormones. Nat. Rev. Cardiol..

[6] Morselli E., Santos R.S., Criollo A., Nelson M.D., Palmer B.F., Clegg D.J. (2017). The Effects of Oestrogens and Their Receptors on Cardiometabolic Health. Nat. Rev. Endocrinol..

[7] Aryan L., Younessi D., Zargari M., Banerjee S., Agopian J., Rahman S., Borna R., Ruffenach G., Umar S., Eghbali M. (2020). The Role of Estrogen Receptors in Cardiovascular Disease. Int. J. Mol. Sci..

[8] Hsu S.-P., Lee W.-S. (2020). Effects of Female Sex Hormones on the Development of Atherosclerosis. Chin. J. Physiol..

[9] Zhang H., Sairam M.R. (2014). Sex Hormone Imbalances and Adipose Tissue Dysfunction Impacting on Metabolic Syndrome; a Paradigm for the Discovery of Novel Adipokines. Horm. Mol. Biol. Clin. Investig..

[10] Cifkova R., Pitha J., Krajcoviechova A., Kralikova E. (2019). Is the Impact of Conventional Risk Factors the Same in Men and Women? Plea for a More Gender-Specific Approach. Int. J. Cardiol..

[11] Huxley R.R., Peters S.A.E., Mishra G.D., Woodward M. (2015). Risk of All-Cause Mortality and Vascular Events in Women versus Men with Type 1 Diabetes: A Systematic Review and Meta-Analysis. Lancet Diabetes Endocrinol..

[12] Martínez D., Castro A., Merino P.M., López P., Lardone M.C., Iñiguez G., Cassorla F., Codner E. (2016). Oestrogen Activity of the Serum in Adolescents with Type 1 Diabetes. Diabet. Med..

[13] Smigoc Schweiger D., Battelino T., Groselj U. (2021). Sex-Related Differences in Cardiovascular Disease Risk Profile in Children and Adolescents with Type 1 Diabetes. Int. J. Mol. Sci..

[14] Szadkowska A., Madej A., Ziółkowska K., Szymańska M., Jeziorny K., Mianowska B., Pietrzak I. (2015). Gender and Age—Dependent Effect of Type 1 Diabetes on Obesity and Altered Body Composition in Young Adults. Ann. Agric. Environ. Med..

[15] Barnes J.N. (2017). Sex-specific Factors Regulating Pressure and Flow. Exp. Physiol..

[16] Freedman R.R., Sabharwal S.C., Desai N. (1987). Sex Differences in Peripheral Vascular Adrenergic Receptors. Circ. Res..

[17] Hart E.C., Charkoudian N., Wallin B.G., Curry T.B., Eisenach J., Joyner M.J. (2011). Sex and Ageing Differences in Resting Arterial Pressure Regulation: The Role of the Β-adrenergic Receptors. J. Physiol..

[18] Faber J.E., Moore S.M., Lucitti J.L., Aghajanian A., Zhang H. (2017). Sex Differences in the Cerebral Collateral Circulation. Transl. Stroke Res..

[19] Sullivan J.C., Rodriguez-Miguelez P., Zimmerman M.A., Harris R.A. (2015). Differences in Angiotensin (1–7) between Men and Women. Am. J. Physiol. Circ. Physiol..

[20] Arnetz L., Rajamand Ekberg N., Alvarsson M. (2014). Sex Differences in Type 2 Diabetes: Focus on Disease Course and Outcomes. Diabetes Metab. Syndr. Obes. Targets Ther..

[21] Hendriks-Balk M., Damianaki A., Theiler K., Polychronopoulou E., Brito W., Pruijm M., Wuerzner G. (2023). Sex Differences in Renal Microcirculation of Hypertensive Patients. J. Hypertens..

[22] Gillis E.E., Sasser J.M., Sullivan J.C. (2016). Endothelin, Sex, and Pregnancy: Unique Considerations for Blood Pressure Control in Females. Am. J. Physiol. Integr. Comp. Physiol..

[23] Mauvais-Jarvis F., Bairey Merz N., Barnes P.J., Brinton R.D., Carrero J.-J., DeMeo D.L., De Vries G.J., Epperson C.N., Govindan R., Klein S.L. (2020). Sex and Gender: Modifiers of Health, Disease, and Medicine. Lancet.

[24] Patel H., Rosengren A., Ekman I. (2004). Symptoms in Acute Coronary Syndromes: Does Sex Make a Difference?. Am. Heart J..

[25] El-Awaisi J., Mitchell J.L., Ranasinghe A., Kalia N. (2023). Interleukin-36 Is Vasculoprotective in Both Sexes despite Sex-Specific Changes in the Coronary Microcirculation Response to IR Injury. Front. Cardiovasc. Med..

[26] Neubauer-Geryk J., Hoffmann M., Wielicka M., Piec K., Kozera G., Brzeziński M., Bieniaszewski L. (2019). Current Methods for the Assessment of Skin Microcirculation: Part 1. Adv. Dermatol. Allergol..

[27] Neubauer-Geryk J., Hoffmann M., Wielicka M., Piec K., Kozera G., Bieniaszewski L. (2019). Current Methods for the Assessment of Skin Microcirculation: Part 2. Adv. Dermatol. Allergol..

[28] Cracowski J., Roustit M. (2016). Current Methods to Assess Human Cutaneous Blood Flow: An Updated Focus on Laser-Based-Techniques. Microcirculation.

[29] Jörneskog G., Brismar K., Fagrell B. (1995). Skin Capillary Circulation Severely Impaired in Toes of Patients with IDDM, with and without Late Diabetic Complications. Diabetologia.

[30] Tibiriçá E., Rodrigues E., Cobas R.A., Gomes M.B. (2007). Endothelial Function in Patients with Type 1 Diabetes Evaluated by Skin Capillary Recruitment. Microvasc. Res..

[31] Neubauer-Geryk J., Kozera G.M., Wolnik B., Szczyrba S., Nyka W.M., Bieniaszewski L. (2013). Decreased Reactivity of Skin Microcirculation in Response to L-Arginine in Later-Onset Type 1 Diabetes. Diabetes Care.

[32] Gasser P., Berger W. (1992). Nailfold Videomicroscopy and Local Cold Test in Type I Diabetics. Angiology.

[33] Kuryliszyn-Moskal A., Dubicki A., Zarzycki W., Zonnenberg A., Górska M. (2011). Microvascular Abnormalities in Capillaroscopy Correlate with Higher Serum IL-18 and SE-Selectin Levels in Patients with Type 1 Diabetes Complicated by Microangiopathy. Folia Histochem. Cytobiol..

[34] Hoffmann M., Neubauer-Geryk J., Wielicka M., Kowaleczko M., Myśliwiec M., Bieniaszewski L. (2019). The Impact of Autoimmune Thyroiditis on Skin Microcirculation in Children with Non-Complicated Type 1 Diabetes Mellitus. Microvasc. Res..

[35] Tooke J. (1986). Microvascular Haemodynamics in Diabetes Mellitus. Clin. Sci..

[36] Tooke J.E., Lins P.E., Ostergren J., Fagrell B. (1985). Skin Microvascular Autoregulatory Responses in Type I Diabetes: The Influence of Duration and Control. Int. J. Microcirc. Clin. Exp..

[37] Neubauer-Geryk J., Wielicka M., Hoffmann M., Myśliwiec M., Bieniaszewski L. (2024). The Impact of Disease Duration on Microcirculatory Dysfunction in Young Patients with Uncomplicated Type 1 Diabetes. Biomedicines.

[38] Triantafyllou A., Anyfanti P., Triantafyllou G., Zabulis X., Aslanidis S., Douma S. (2016). Impaired Metabolic Profile Is a Predictor of Capillary Rarefaction in a Population of Hypertensive and Normotensive Individuals. J. Am. Soc. Hypertens..

[39] Barchetta I., Riccieri V., Vasile M., Stefanantoni K., Comberiati P., Taverniti L., Cavallo M.G. (2011). High Prevalence of Capillary Abnormalities in Patients with Diabetes and Association with Retinopathy. Diabet. Med..

[40] Fahrig C., Breitinger L., Heidrich H. (2000). Vital Capillary Microscopic Findings in the Nailfold of Patients with Diabetes Mellitus. VASA.

[41] Buss C., Maranhão P.A., de Souza M., Bouskela E., Kraemer-Aguiar L.G. (2020). Obesity Blunts Cephalic-Phase Microvascular Responses to Food. Physiol. Behav..

[42] Rendell M., Bergman T., O’Donnell G., Drobny E., Borgos J., Bonner R.F. (1989). Microvascular Blood Flow, Volume, and Velocity Measured by Laser Doppler Techniques in IDDM. Diabetes.

[43] Hauser C.J., Klein S.R., Mehringer C.M., Appel P., Shoemaker W.C. (1984). Assessment of Perfusion in the Diabetic Foot by Regional Transcutaneous Oximetry. Diabetes.

[44] Bauersachs R., Shaw S., Zeidler A., Meiselman H. (1989). Red Blood Cell Aggregation and Blood Viscoelasticity in Poorly Controlled Type 2 Diabetes Mellitus. Clin. Hemorheol. Microcirc..

[45] Rendell M., Fox M., Knox S., Lastovica J., Kirchain W., Meiselman H.J. (1991). Effects of Glycemic Control on Red Cell Deformability Determined by Using the Cell Transit Time Analyzer. J. Lab. Clin. Med..

[46] Boulton A.J.M., Scarpello J.H.B., Ward J.D. (1982). Venous Oxygenation in the Diabetic Neuropathic Foot: Evidence of Arteriovenous Shunting?. Diabetologia.

[47] Ladurner R., Küper M., Königsrainer I., Löb S., Wichmann D., Königsrainer A., Coerper S., Beckert S. (2010). Predictive Value of Routine Transcutaneous Tissue Oxygen Tension (TcpO2) Measurement for the Risk of Non-Healing and Amputation in Diabetic Foot Ulcer Patients with Non-Palpable Pedal Pulses. Med. Sci. Monit. Int. Med. J. Exp. Clin. Res..

[48] Koch C., Chauve E., Chaudru S., Le Faucheur A., Jaquinandi V., Mahé G. (2016). Exercise Transcutaneous Oxygen Pressure Measurement Has Good Sensitivity and Specificity to Detect Lower Extremity Arterial Stenosis Assessed by Computed Tomography Angiography. Medicine.

[49] Nishio H., Minakata K., Kawaguchi A., Kumagai M., Ikeda T., Shimizu A., Yokode M., Morita S., Sakata R. (2016). Transcutaneous Oxygen Pressure as a Surrogate Index of Lower Limb Amputation. Int. Angiol..

[50] Moosa H.H., Peitzman A.B., Makaroun M.S., Webster M.W., Steed D.L. (1988). Transcutaneous Oxygen Measurements in Lower Extremity Ischemia: Effects of Position, Oxygen Inhalation, and Arterial Reconstruction. Surgery.

[51] Papa G., Spazzapan L., Pangos M., Delpin A., Arnež Z.M. (2014). Compared to Coverage by STSG Grafts Only Reconstruction by the Dermal Substitute Integra^®^ plus STSG Increases TcPO2 Values in Diabetic Feet at 3 and 6 Months after Reconstruction. J. Ital. Assoc. Hosp. Surg..

[52] Neubauer-Geryk J., Wielicka M., Kozera G.M., Brandt-Varma A., Wołoszyn-Durkiewicz A., Myśliwiec M., Bieniaszewski L. (2021). Skin Oxygenation Impairment Is Associated with Increased Total Cholesterol Level in Children with Short-Lasting Type 1 Diabetes Mellitus. Adv. Dermatol. Allergol..

[53] Neubauer-Geryk J., Wielicka M., Myśliwiec M., Zorena K., Bieniaszewski L. (2023). The Relationship between TNF-a, IL-35, VEGF and Cutaneous Microvascular Dysfunction in Young Patients with Uncomplicated Type 1 Diabetes. Biomedicines.

[54] Libman I., Haynes A., Lyons S., Pradeep P., Rwagasor E., Tung J.Y., Jefferies C.A., Oram R.A., Dabelea D., Craig M.E. (2022). Clinical Practice Consensus Guidelines 2022: Definition, Epidemiology, and Classification of Diabetes in Children and Adolescents. Pediatr. Diabetes.

[55] Marshall W.A., Tanner J.M. (1970). Variations in the Pattern of Pubertal Changes in Boys. Arch. Dis. Child..

[56] Marshall W.A., Tanner J.M. (1969). Variations in Pattern of Pubertal Changes in Girls. Arch. Dis. Child..

[57] ElSayed N.A., Aleppo G., Bannuru R.R., Bruemmer D., Collins B.S., Ekhlaspour L., Gaglia J.L., Hilliard M.E., Johnson E.L., Khunti K. (2024). Diagnosis and Classification of Diabetes: Standards of Care in Diabetes—2024. Diabetes Care.

[58] Dyck P.J. (1988). Detection, Characterization, and Staging of Polyneuropathy: Assessed in Diabetics. Muscle Nerve.

[59] Falkner B., Gidding S.S., Baker-Smith C.M., Brady T.M., Flynn J.T., Malle L.M., South A.M., Tran A.H., Urbina E.M. (2023). Pediatric Primary Hypertension: An Underrecognized Condition: A Scientific Statement from the American Heart Association. Hypertension.

[60] Leenstra B., Wijnand J., Verhoeven B., Koning O., Teraa M., Verhaar M.C., de Borst G.J. (2020). Applicability of Transcutaneous Oxygen Tension Measurement in the Assessment of Chronic Limb-Threatening Ischemia. Angiology.

[61] Fife C.E., Smart D.R., Sheffield P.J., Hopf H.W., Hawkins G., Clarke D. (2009). Transcutaneous Oximetry in Clinical Practice: Consensus Statements from an Expert Panel Based on Evidence. Undersea Hyperb. Med..

[62] Chambliss K.L., Shaul P.W. (2002). Estrogen Modulation of Endothelial Nitric Oxide Synthase. Endocr. Rev..

[63] Burkhardt M., Slotta J.E., Garcia P., Seekamp A., Menger M.D., Pohlemann T. (2007). The Effect of Estrogen on Hepatic Microcirculation after Ischemia/Reperfusion. Int. J. Color. Dis..

[64] Campisi R., Nathan L., Pampaloni M.H., Schöder H., Sayre J.W., Chaudhuri G., Schelbert H.R. (2002). Noninvasive Assessment of Coronary Microcirculatory Function in Postmenopausal Women and Effects of Short-Term and Long-Term Estrogen Administration. Circulation.

[65] Worboys S., Kotsopoulos D., Teede H., McGrath B., Davis S.R. (2001). Evidence That Parenteral Testosterone Therapy May Improve Endothelium-Dependent and -Independent Vasodilation in Postmenopausal Women Already Receiving Estrogen. J. Clin. Endocrinol. Metab..

[66] Kao W.-L., Sun C.-W. (2015). Gender-Related Effect in Oxygenation Dynamics by Using Far-Infrared Intervention with Near-Infrared Spectroscopy Measurement: A Gender Differences Controlled Trial. PLoS ONE.

[67] Reckelhoff J.F. (2001). Gender Differences in the Regulation of Blood Pressure. Hypertension.

[68] Grau M., Cremer J.M., Schmeichel S., Kunkel M., Bloch W. (2018). Comparisons of Blood Parameters, Red Blood Cell Deformability and Circulating Nitric Oxide between Males and Females Considering Hormonal Contraception: A Longitudinal Gender Study. Front. Physiol..

[69] White R.E. (2002). Estrogen and Vascular Function. Vascul. Pharmacol..

[70] Charkoudian N., Hart E.C.J., Barnes J.N., Joyner M.J. (2017). Autonomic Control of Body Temperature and Blood Pressure: Influences of Female Sex Hormones. Clin. Auton. Res..

[71] Andrade L.E.C., Gabriel A., Assad R.L., Ferrari A.J.L., Atra E. (1990). Panoramic Nailfold Capillaroscopy: A New Reading Method and Normal Range. Semin. Arthritis Rheum..

[72] Terreri M.T.R.A., Andrade L.E.C., Puccinelli M.L., Hilário M.O.E., Goldenberg J. (1999). Nail Fold Capillaroscopy: Normal Findings in Childrenand Adolescents. Semin. Arthritis Rheum..

[73] Bogusz-Górna K., Polańska A., Dańczak-Pazdrowska A., Żaba R., Sumińska M., Fichna P., Kędzia A. (2023). Non-Invasive Detection of Early Microvascular Changes in Juveniles with Type 1 Diabetes. Cardiovasc. Diabetol..

[74] Hegyi J., Hegyi V., Messer G., Arenberger P., Ruzicka T., Berking C. (2009). Confocal Laser-scanning Capillaroscopy: A Novel Approach to the Analysis of Skin Capillaries In Vivo. Ski. Res. Technol..

[75] Heimhalt-El Hamriti M., Schreiver C., Noerenberg A., Scheffler J., Jacoby U., Haffner D., Fischer D.-C. (2013). Impaired Skin Microcirculation in Paediatric Patients with Type 1 Diabetes Mellitus. Cardiovasc. Diabetol..

[76] Stupin A., Stupin M., Baric L., Matic A., Kolar L., Drenjancevic I. (2019). Sex-Related Differences in Forearm Skin Microvascular Reactivity of Young Healthy Subjects. Clin. Hemorheol. Microcirc..

[77] Orenstein A., Mazkereth R., Tsur H. (1988). Mapping of the Human Body Skin with the Transcutaneous Oxygen Pressure Method. Ann. Plast. Surg..

[78] Dooley J., King G., Slade B. (1997). Establishment of Reference Pressure of Transcutaneous Oxygen for the Comparative Evaluation of Problem Wounds. Undersea Hyperb. Med. J. Undersea Hyperb. Med. Soc..

[79] Rodrigues L.M., Contreiras Pinto P., Leal A. (2001). Transcutaneous Flow Related Variables Measured In Vivo: The Effects of Gender. BMC Dermatol..

[80] Dowd G.S.E., Linge K., Bentley G. (1983). The Effect of Age and Sex of Normal Volunteers upon the Transcutaneous Oxygen Tension in the Lower Limb. Clin. Phys. Physiol. Meas..

[81] Rossi M., Pistelli F., Pesce M., Aquilini F., Franzoni F., Santoro G., Carrozzi L. (2014). Impact of Long-Term Exposure to Cigarette Smoking on Skin Microvascular Function. Microvasc. Res..

[82] Lenasi H., Štrucl M. (2010). Regular Physical Activity Alters the Postocclusive Reactive Hyperemia of the Cutaneous Microcirculation. Clin. Hemorheol. Microcirc..

[83] Wang J., Lan C., Chen S., Wong M. (2002). Tai Chi Chuan Training Is Associated with Enhanced Endothelium-Dependent Dilation in Skin Vasculature of Healthy Older Men. J. Am. Geriatr. Soc..

